# Evaluating the impact of parameter variability on O_2_ control and model robustness in modified atmosphere storage for fresh produce

**DOI:** 10.1038/s41598-025-99854-4

**Published:** 2025-05-12

**Authors:** Yogesh B. Kalnar, Cornelia Weltzien, Pramod V. Mahajan

**Affiliations:** 1 Leibniz Institute for Agricultural Engineering and Bioeconomy (ATB), Potsdam, Germany; 2 Chair of Agromechatronics, Technische Universität Berlin, Straße des 17 Juni 135, Berlin, Germany; 3 Indian Council of Agricultural Research-Central Institute of Post-Harvest Engineering and Technology, Ludhiana, India

**Keywords:** Respiration, Gases, Fresh produce, Modelling, Sensor, Controller, Respiratory system models, Enzyme mechanisms

## Abstract

The effects of variation in product respiration, supply chain temperature, gas diffusion, product quantity, and storage volume on gas control in fresh produce storage remain unexplored. This study aimed to evaluate the impact of such parameters on O_2_ control, using broccoli as a case study under dynamic temperature profiles ranging from 1 °C to 20 °C. Sensitivity analysis of each parameter was performed using Monte Carlo simulations and one-factor-at-a-time method and the results were experimentally validated. The blower ON frequency (BOF) needed for O_2_ control exhibited a mean of 47.8 ± 3.7 s, illustrating variability due to model parameter uncertainties. The product weight and respiration rate were the most influential parameters affecting the BOF. These parameters collectively accounted for over 80% of the BOF variability, highlighting the importance of prioritizing and incorporating non-linear relationships and parameter interactions for model robustness. Temperature variations affected BOF and respiration rates while maintaining overall O_2_ and CO_2_ concentrations stable over a longer period. However, O_2_ and CO_2_ concentrations exhibited temporary fluctuations because, at higher temperatures, the blower operated for longer durations compared to lower temperatures, leading to more pronounced O_2_ fluctuations. The results of the validation experiment, using a 70-litre box containing 16 kg of broccoli, confirmed the stability of the model in effectively handling the parameters variation while maintaining the O_2_ concentration at 3.5 ± 0.5% and CO_2_ at 15.3 ± 1% during storage and transport of the fresh produce.

**Supplementary Information:**

The online version contains supplementary material available at 10.1038/s41598-025-99854-4.

## Introduction

To mitigate post-harvest losses, efficient supply chains and practices are crucial. While modified atmosphere (MA) storage boxes with gas-permeable membranes are effective, they are primarily suitable for low temperatures (3–6 °C) due to limited gas permeability. The designed package may fail to achieve its true potential under unintended conditions e.g. temperature abuse, which is often met in the cold chain. It is the main reason, which causes quality degradation of fresh produce during transport and storage^1,2^. Temperature fluctuations in the produce supply chain may disrupt the balance between the package’s gas transmission rates and the physiology of produce, mainly respiration rate, which affects the target atmosphere^3^ and depends on the degree and direction of temperature deviation^4^. Temperature significantly influences the product respiration rate^5^ than the package gas transmission rate, challenging the maintenance of consistent MA during its storage^6–8^.

The MA systems for fruit and vegetable containers also include the automatic air freshening system developed by Thermo King, Bloomington, Minnesota, United States. This system is described as semi-active for maintaining MA conditions during storage and transport. It allows fresh air ventilation after loading until the cargo generates the required concentration of CO_2_. The ventilation rate is adjusted based on CO_2_ concentration, ventilation reduced when concentrations are below the set threshold and increased when the desired CO_2_ concentration is achieved. Controller presets for carriage temperatures and ventilation settings for various fresh produce types were preloaded for ease of use^9^. The Transfresh System, Salinas, California, United States also known as modified Techtrol, was one of the pioneering commercial systems. It relies on a tightly sealed container equipped with purge ports for initial atmosphere development, a door curtain, and an O_2_ valve controller. A dedicated controller continuously monitored O_2_ and CO_2_ concentration using preinstalled sensors. When the O_2_ concentration drops too low, the controller opens the valve to introduce outside air. For CO_2_ control, fresh air ventilation was used to increase CO_2_ concentration (by opening a valve), or when low O_2_ and CO_2_ concentrations were needed, adsorption with hydrated lime was employed^10^. However, such systems require gas sensors and extensive control mechanisms, making them an economically unviable option for transporting smaller quantities of fruit and vegetables.

For small-scale storage systems Jo et al.^11^ developed a mathematical model for the operation of gas control valves placed in the perforated tube. They dynamically controlled the gas composition in MA storage containers based on O_2_ concentration, ensuring desired gas compositions for green pepper and spinach under varying temperature conditions. Building on this, subsequent studies^12–14^ introduced adaptive control mechanisms and iterative operations to respond to the respiration of fresh produce in real-time. In parallel, devices and methods for monitoring and controlling fresh produce respiration during transport have been developed by Savur et al.^15^; Savur et al.^16^, Savur et al.^17^ and Alzuabi et al.^18^. These systems include sensors, a refrigeration unit, gas inlets/outlets and a controller. They maintained optimal storage conditions during transport. However, challenges such as cost, size and reliance on gas sensors for feedback control remain, and simpler, more robust and cost-effective solutions are needed.

A gas control system using forced air diffusion was developed to regulate gas concentration in storage containers of sweet cherries in the study by Keshri et al.^19^. Experimental determination of forced and natural air diffusion coefficients facilitated the estimation of blower operation required to maintain desired gas concentration at different storage temperatures. As an extension of this work Jalali, et al.^20^ modelled the storage environment for sweet cherries and controlled the CO_2_ concentration in the containers with a control system. However, manual adjustment of the blower operation during temperature changes posed operational challenges, highlighting the need for automated solutions in the transport of fresh produce. Extending upon this Kalnar et al.^21^ automated the blower operation using time-temperature mapping and validated the model output with broccoli storage as a case study. They used the term blower ON frequency (BOF) which is defined as the time in seconds required for the blower system to activate every hour to regulate the O_2_ concentration within the storage box.

The influence of the variations in produce respiration and temperature fluctuation^22^, also the potential changes in the gas diffusion through the storage box and the amount of product, and storage volume has not been done yet. Therefore, this work aimed to evaluate the impact of variation in key input parameters on BOF and O_2_ concentration in the storage box. Key factors include product weight, respiration rate, gas diffusion rate and size of storage box. Broccoli was used as a case study to evaluate the impact of variability on O_2_ control under fluctuating temperature profiles.

## Materials and methods

### Modified atmosphere storage box

**Fig. 1:**
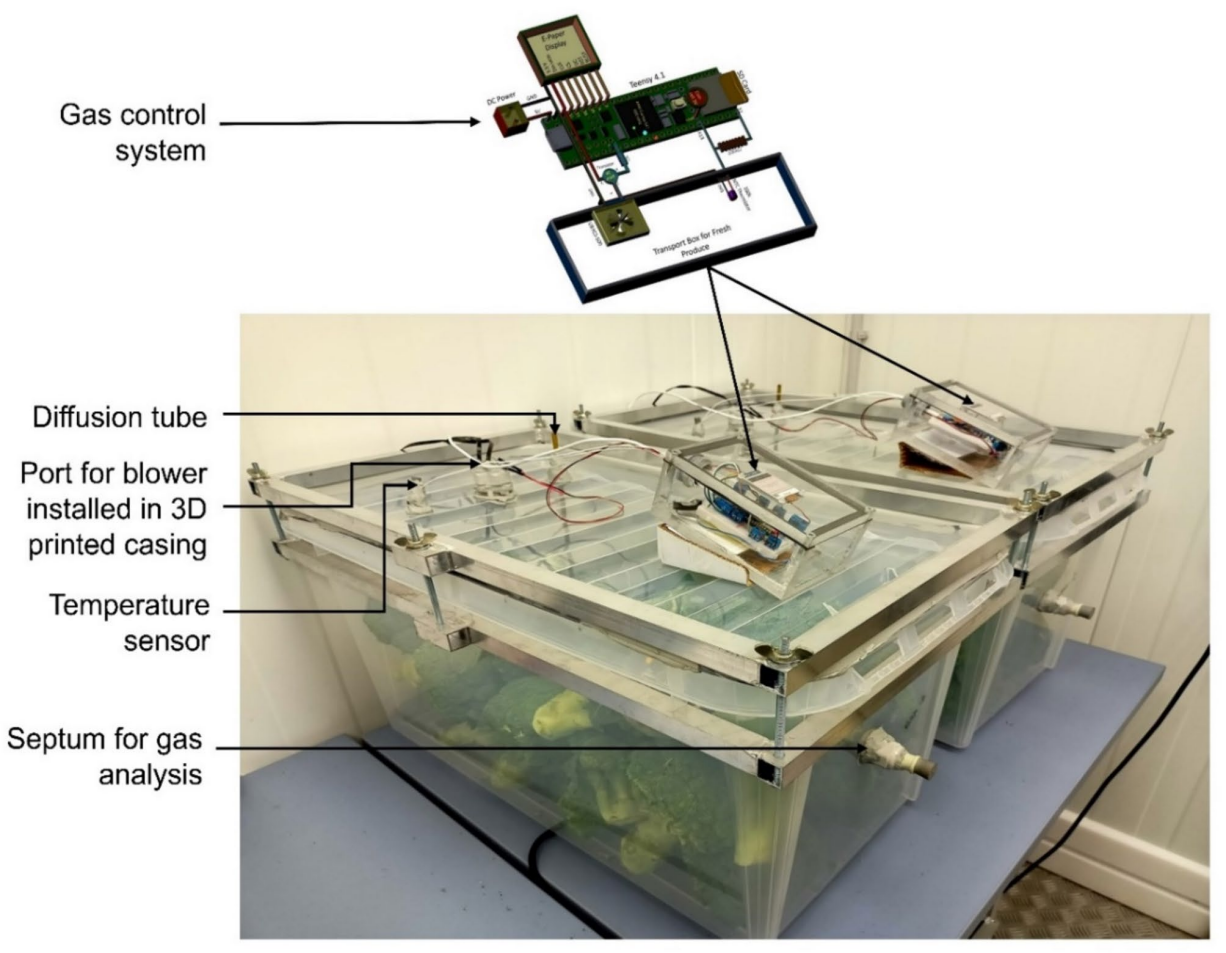
Storage boxes showing diffusion tube, air blower and the gas control system used for broccoli storage.

The experimental set-up for storing 16 kg of broccoli used a 70-litre transparent box with a lid, bought from the local retailer (Fig. 1). The rubber gasket around the lid ensured its airtightness. The lid of the box was equipped with an air blower (model UB3C3-500, Sunonwealth Electric Machine Industry, Kaohsiung, Taiwan), sized as 12 mm x 12 mm x 3 mm and with automatic restart capability when blocked by any foreign material. This blower was connected to a Teensy 4.1 controller, which integrated a mathematical model incorporating real-time temperature data. The blower was securely housed in a 3D-printed housing made from polylactic acid filament (EasyFil, Form Futura, The Netherlands), selected for its impact resistance, flow behaviour and interlayer adhesion properties. Moreover, a diffusion tube measuring 250 mm in length and with an internal diameter of 6 mm was inserted through a sealed opening in the box lid to restrict the entry of air but enable air exchange through it when the blower was activated. The diffusion tube remained open at all times, regardless of blower operation. However, due to its smaller diameter and extended length, passive air exchange through the tube was negligible^19^. Consequently, during blower operation, air entered through the blower and exited via the tube. An additional rubber septum (Suba-Seal septa, Sigma-Aldrich Co LLC, United Kingdom) was provided for gas sampling by a headspace gas analyser (CheckMate3, MOCON Europe A/S (Dansensor), Ringsted, Denmark) .In the gas control system setup, a Teensy 4.1 microcontroller (PJRC.COM, LLC., Oregon, USA) was used to regulate the blower and thus O_2_ concentration inside the box. The gas control system used a 1.54-inch ePaper display (E-paper E-Ink (B), Waveshare 13338, China) to show real-time parameters from the box such as temperature, and BOF for one hour cycle. A radial mini air blower (UB3C3-500) to purge the fresh air in the box, a 100 K thermistor with a negative temperature coefficient for temperature measurement of the produce placed in the box, and onboard data storage with real-time clock functionality were used. The Teensy 4.1, chosen for its advanced capabilities, composed hourly temperature checks, calculated blower operation time, logged data and activated the air blower for precise control of the O_2_ concentration inside the box.

### Mathematical formulation for gas control

**Fig. 2:**
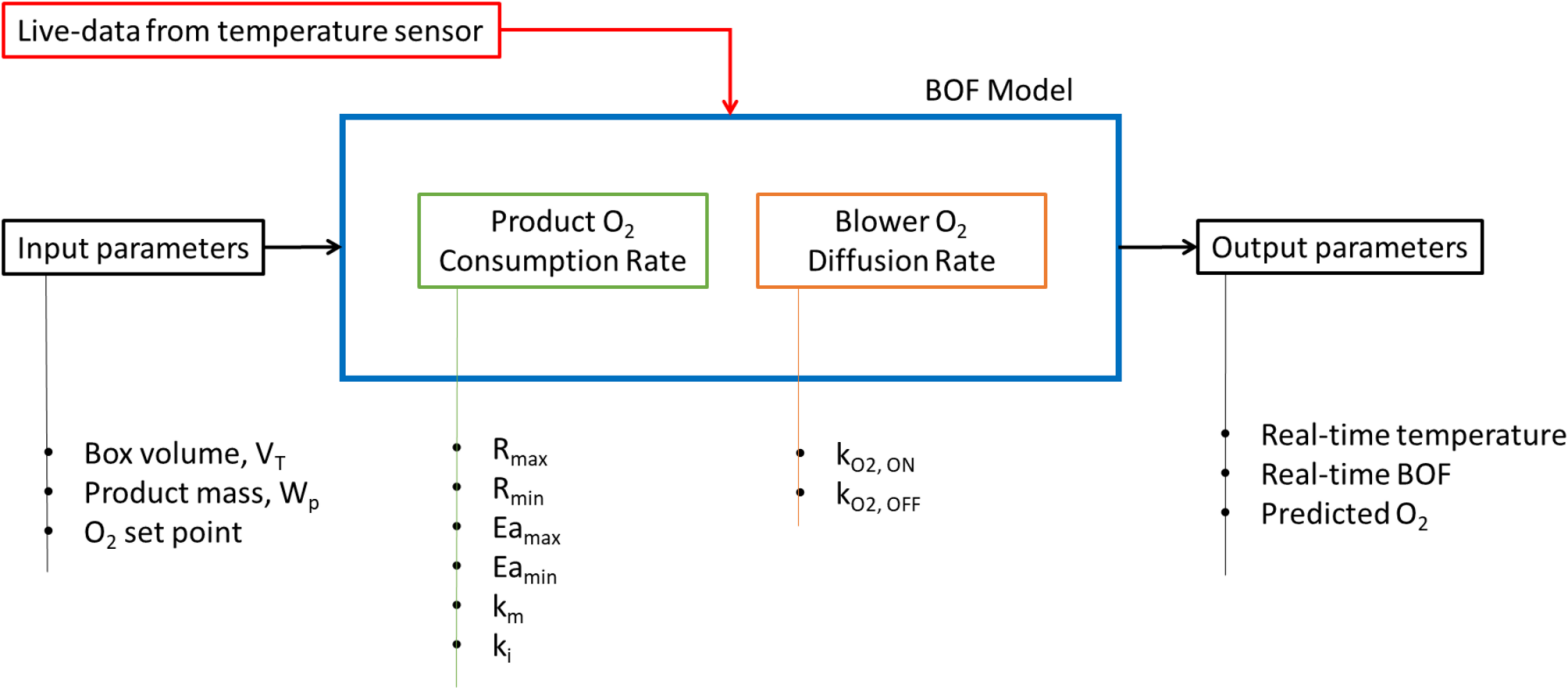
Model input and output parameters for O_2_ control in the storage box, also highlighting BOF model used real-time temperature values of the produce stored in the box for BOF estimation.

The mathematical formulation for the gas control system used in this study is based on a modified BOF model previously reported by Kalnar et al.^21^. This model integrates key factors such as product O_2_ consumption rate, O_2_ diffusion rate, storage box volume and product mass (Fig. 2). The process of O_2_ diffusion through the blower was categorised into two distinct phases: the forced phase occurring when the blower was activated, and the natural phase when the blower was deactivated. The BOF represents the total time (in seconds) required to keep the blower ON during a one-hour cycle to maintain O_2_ set point to 3%. It is determined using Eq. (1).

1 $$\:BOF=\:\left[\:\frac{\frac{d{V{o}_{2}}_{rr,set}}{dt}\:-\:3600\:\:\frac{d{V{o}_{2}}_{b{l.}_{OFF}}}{dt}}{\frac{d{V{o}_{2}}_{b{l.}_{ON}}}{dt}\:-\:\frac{d{V{o}_{2}}_{b{l.}_{OFF}}}{dt}}\right]$$

The terms used in Eq. (1) $\:\frac{d{V{o}_{2}}_{rr,set}}{dt}\:$ is the rate of O_2_ consumed (mL kg^− 1^ h^− 1^) by the broccoli was computed using the Eq. (2), which is already integrated with an Arrhenius equation for temperature effect on respiration rate.

2 $$\:\frac{d{V{o}_{2}}_{rr,set}}{dt}=\:\left[\frac{{\text{R}}_{\text{m}\text{a}\text{x}}\:{e}^{\left(\:\frac{{{-E}_{a}}_{max}}{{\text{R}}_{\text{g}}\:\left(\:T+273\right)}\right)}{O}_{{2}_{set}}}{{{k}_{m}}_{\:}+\left(1+\frac{{CO}_{2}}{{k}_{i}}\right){O}_{{2}_{set}}}+{{\text{R}}_{\text{m}\text{i}\text{n}}}_{\:}{e}^{\left(\:\:\frac{{{-E}_{a}}_{min}}{{\text{R}}_{\text{g}}\:\left(T+273\right)}\right)}\right]\:{W}_{p}\:\frac{1}{3600}$$

where $\:{{k}_{m}}_{\:}$ and $\:{k}_{i}$are the Michaelis-Menten and inhibition constant in %,$\:{\:\text{R}}_{\text{m}\text{a}\text{x}}$ and $\:{\text{R}}_{\text{m}\text{i}\text{n}}$ are the pre-exponential factors^23^ while $\:{{E}_{a}}_{max}$ and $\:{{E}_{a}}_{min}$ are the activation energies (J mole^− 1^), under the conditions of abundant and low O_2_ availability, respectively. To avoid unusually high values of the pre-exponential factor, it is recommended to use the Arrhenius equation with a reference temperature. The $\:{O}_{{2}_{set}}\:$and $\:{CO}_{2}\:$are the set point (3%) for O_2_ and CO_2_ (15%), respectively. *R*_*g*_ is the gas constant (8.314 J K^− 1^ mol^− 1^), *T* is the temperature (°C), and $\:{W}_{p}$ is the weight (kg) of the broccoli in the storage box.

The term $\:{V{o}_{2}}_{b{l.}_{ON}}$ is the volume of O_2_ (mL) diffused into the box containing 16 kg ($\:{W}_{p})\:$broccoli during the forced phase (i.e. when the blower ON) was calculated by applying Eq. (3) for a storage box with total volume (V_T_) 0.07 m^3^ and considering the density (D_p_) of the broccoli as 1050 kg/m^3^. Free volume/head space was considered for estimating the required volume of air during blower ON and OFF time.

3 $$\:\frac{d{V{o}_{2}}_{b{l.}_{ON}}}{dt}=\:\frac{\left({\text{V}}_{T}-\frac{{\text{W}}_{\text{p}}}{{\text{D}}_{\text{p}}}\right)}{0.36\:}\left[21+{\left({O}_{{2}_{set}}-21\right)\:e}^{\left(\:\frac{-{k}_{{\text{O}}_{{2}_{\text{O}\text{N}}}\:}}{\left({\text{V}}_{T}-\frac{{\text{W}}_{\text{p}}}{{\text{D}}_{\text{p}}}\right)}\:t\right)}-{O}_{{2}_{set}}\right]$$

Where $\:{V{o}_{2}}_{b{l.}_{OFF}}$ is the volume of O_2_ (mL) diffused into the box during the natural phase (i.e. when the blower OFF) through diffusion tube and it was estimated using Eq. (4), for time t (s).

4 $$\:\frac{d{V{o}_{2}}_{b{l.}_{OFF}}}{dt}=\:\frac{\left({\text{V}}_{T}-\frac{{\text{W}}_{\text{p}}}{{\text{D}}_{\text{p}}}\right)}{0.36\:}\left[21+{\left({O}_{{2}_{set}}-21\right)\:e}^{\left(\:\frac{-{k}_{{\text{O}}_{{2}_{\text{O}\text{F}\text{F}}}\:}}{\left({\text{V}}_{T}-\frac{{\text{W}}_{\text{p}}}{{\text{D}}_{\text{p}}}\right)}\:t\right)}-{O}_{{2}_{set}}\right]$$

where $\:{k}_{{\text{O}}_{{2}_{\text{O}\text{N}}}}$and $\:{k}_{{\text{O}}_{{2}_{\text{O}\text{f}\text{f}}}}$ (m^3^ s^− 1^) are the diffusion coefficients of the air when the blower was ON and OFF respectively. The numerical factor 21 represents the O_2_ concentration in ambient air (21%). This term takes into account the difference between the set concentration ($\:{O}_{{2}_{set}}$) and the O_2_ available under normal atmospheric conditions. The factor 0.36 is a unit conversion factor used to scale the equation appropriately. This factor resulted from a combination of empirical adjustments/unit conversions. The model (Eq. 1) was implemented directly into a Teensy microcontroller system. This enabled real-time calculations of the BOF in every one-hour cycle. At the start of each cycle, the microcontroller utilised temperature data from a sensor installed in the storage box to determine broccoli temperature. Using this information, it calculated the BOF instantaneously and immediately applied the result to control the blower’s operation. This hourly iterative process continued throughout the storage period, with the microcontroller continuously monitoring the internal temperature and dynamically adjusting the BOF to maintain the O_2_ set point in the box.

### Assessing variability in the gas control parameters

The effectiveness of the gas control model depends on the variability of key parameters influenced by product characteristics, storage conditions, and gas diffusion. This section addresses the model’s robustness under realistic conditions, including variations in product respiration rates, diffusion coefficients, and storage configurations. A systematic approach was used to assess the sensitivity of these parameters and their impact on maintaining O_2_ set point during storage. The key sources of variability were categorized into three domains:

Product variability:

- Pre-exponential factors ($\:{R}_{max}\:\:$and $\:{R}_{min}$)
- Activation energies ($\:{{E}_{a}}_{max}\:$and $\:{{E}_{a}}_{min}$)
- Michaelis-Menten constant ($\:{{k}_{m}}_{\:}$)
- O_2_ inhibition constant ($$\:{k}_{i}$$)

Diffusion variability:

- O_2_ diffusion rate with the blower ON ($\:{k}_{{\text{O}}_{{2}_{\text{O}\text{N}}}}$)
- O_2_ diffusion rate with the blower OFF ($\:{k}_{{\text{O}}_{{2}_{\text{O}\text{F}\text{F}}}}$)

Variability in storage parameters:

- Product weight ($$\:{W}_{p}$$)
- Storage box free volume ($\:{V}_{T}$)

The base values of the model parameters (Table 1) were taken from Kalnar et al.^21^ and the systemic variations were introduced to determine their effect on O_2_ concentration and BOF.

**Table 1:** Parameter values with their abbreviation and units used for variability analysis^21^.

Parameter	Abbreviation	Unit	Base value	Absolute error	% error
Activation energy when O_2_ is abundantly available	$\:{{E}_{a}}_{max}$	J mol^− 1^	8.63E + 04	22.9	0.03
Activation energy under low O_2_ conditions	$\:{{E}_{a}}_{min}$	J mol^− 1^	1.03E + 05	43.6	0.04
Pre-exponential factor when O_2_ is abundantly available	$\:{{R}_{max}}_{\:}$	mL kg^− 1^ h^− 1^	2.76E + 17	1.38E + 16	5
Pre-exponential factor under low O_2_ conditions	$\:{{R}_{min}}_{\:}$	mL kg^− 1^ h^− 1^	9.90E + 19	4.95E + 18	5
Michaelis-Menten constant	$\:{{k}_{m}}_{\:}$	%	19.6	2.4	12.3
Inhibition constant	$\:{k}_{i}$	%	8.07	2.2	27.3
Diffusion coefficient of O_2_ when the blower OFF	$\:{k}_{{\text{O}}_{{2}_{\text{O}\text{F}\text{F}}}}$	m^3^/s	2.00E-08	1.00E-09	5
Diffusion coefficient of O_2_ when the blower ON	$\:{k}_{{\text{O}}_{{2}_{\text{O}\text{N}}}}$	m^3^/s	2.28E-05	6.00E-08	0.26
Weight of the produce	$\:{W}_{p}$	kg	16	0.8	5
Total volume of the storage box	$\:{\text{V}}_{T}$	m^3^	0.07	0.0035	5

#### Variability analysis by Monte Carlo method

The Monte Carlo method was used to evaluate the model parameter’s sensitivity and interaction effects on BOF because of its statistical robustness in assessing the cumulative impact of parameter-inherent uncertainties. The Monte Carlo simulation for the BOF model was performed using Python 3.13.0 along with the libraries for statistical analysis and data visualisation. *NumPy* 2.1.3 was used for numerical calculations, while *SciPy* 1.14.1 facilitated scientific computing and optimisation. *Pandas* 2.2.3 was used for data handling and analysis, ensuring efficient manipulation of data. For visualisation, *Matplotlib* 3.9.2 and *Seaborn* 0.13.2 was used to generate plots. The standard deviations for each parameter were estimated from computational simulations using experimentally determined base values and respective absolute/percentage errors (Table 1). The parameters were assumed to follow an independent normal distribution, allowing the simulations to account for realistic variations in the input parameters. Normal distribution was considered for the study because, in a real supply chain, parameters related to product, diffusion and storage may fluctuate slightly around their true value. A total of 10,000 iterations were used to evaluate the variability. In each iteration, the input parameters were randomly sampled from their respective probability distributions to calculate the BOF using Eq. (1). Sensitivity and interaction analysis was performed to identify the most influential parameters influencing BOF by changing each parameter within its percentage error range. Sensitivity ranges and rankings were derived to quantify each parameter’s relative influence on the BOF. Interaction effects were visualized using scatter plots and correlation matrices, which highlighted the non-linear relationships and potential redundancies in the contributions of the parameters. The analysis was performed using a Python script with the fixed storage parameters such as temperature (T) at 10 °C, $\:{CO}_{2}$ concentration at 15%, O_2_ set point ($\:{O}_{{2}_{set}}$) at 3%. These storage parameters were chosen because they are optimal for broccoli storage, as reported by Fernandez-Leon et al.^24^ and Kalnar et al.^21^.

#### One-at-a-time approach

Each parameter was varied individually (± standard error or ± 5%) with positive (addition of standard error in base value) and negative (subtracting standard error from base value) variation from the base value while holding all other parameters constant (at base value), as per method reported by Jurij et al.^25^. New O_2_ concentration values were predicted for each parameter considering values given in Table 1 and using the equation reported by Keshri et al.^19^ and Jalali, et al.^20^. The values of predicted O_2_ concentration were used to evaluate the impact of variability. This approach provided insights into the independent sensitivity of each model parameter but did not account for interactions between the parameters. All parameters were varied simultaneously to address interactions, allowing both positive and negative variations. This comprehensive analysis revealed the combined effects of parameter variability on O_2_ control and helped identify the most sensitive parameters.

The sensitivity in O_2_ concentration $\:\left([O_2]\right)$ when parameters change from its baseline values were quantified as:

$$\:{\Delta\:}[O_2]={[O_2]}_{\text{v}\text{a}\text{r}\text{i}\text{e}\text{d}}\text{}-{[O_2]}_{\text{b}\text{a}\text{s}\text{e}\text{l}\text{i}\text{n}\text{e}}$$​

where, $\:{[O_2]}_{\text{v}\text{a}\text{r}\text{i}\text{e}\text{d}}$​ is the O_2_ concentration computed with the modified parameter estimated considering positive and negative variations and $\:{[O_2]}_{\text{b}\text{a}\text{s}\text{e}\text{l}\text{i}\text{n}\text{e}}$​ is the concentration under baseline value of that parameter.

To incorporate combined variability, a multivariable sensitivity analysis was performed as:

$$\:{{\Delta\:}[O_2]}_{\text{c}\text{o}\text{m}\text{b}\text{i}\text{n}\text{e}\text{d}}=f({R}_{max}\:,\:{R}_{min}\:,{k}_{m},{k}_{i}\:,\:{k}_{{\text{O}}_{{2}_{\text{O}\text{F}\text{F}}}},\:{k}_{{\text{O}}_{{2}_{\text{O}\text{N}}}},\:{W}_{p},{{E}_{a}}_{max},\:{{E}_{a}}_{min},{V}_{T})\:$$

where, $\:f$ represents the model function integrating all parameters with positive and negative variations.

### Experimental validation

Fresh broccoli was sourced from the local supplier to validate the developed gas control system and its predictive BOF model (Werder Frucht GmbH, Groß Kreutz, Germany). The broccoli was packed inside the storage boxes as described in Sect. 2.1. The NTC thermistor connected with the controller was inserted into the core of the broccoli floret within each box. This provided real-time temperature data, which was used by the microcontroller to calculate the BOF based on the model equations dynamically.

Two boxes equipped with a gas control system were placed inside a cold storage facility (Frigotech GmbH, Germany). This facility allowed precise temperature adjustments to simulate the temperature variations experienced in the actual supply chain of broccoli as reported by Cantwell & Suslow^26^. The simulated temperature profile included unit operations such as harvesting in the field and transportation (20 °C), precooling (7 °C), transport to the warehouse (3 °C), storage at the warehouse (1 °C), transport to the distribution centre (5 °C), and storage at the distribution centre (10 °C ). These profiles were mimicked in the walk-in cooling room and the actual measured temperature was used for real-time BOF calculations. Temperature variations influenced the product respiration model parameters, which were addressed with the Arrhenius-type equation. The headspace O_2_ and CO_2_ concentrations in the boxes were sampled at regular intervals using a headspace gas analyser. This data provided insights into the performance of the gas control system over the storage period and was used to assess the model’s accuracy in maintaining the desired O_2_ concentration.

## Results and discussion

### Variability analysis of BOF

**Fig. 3:**
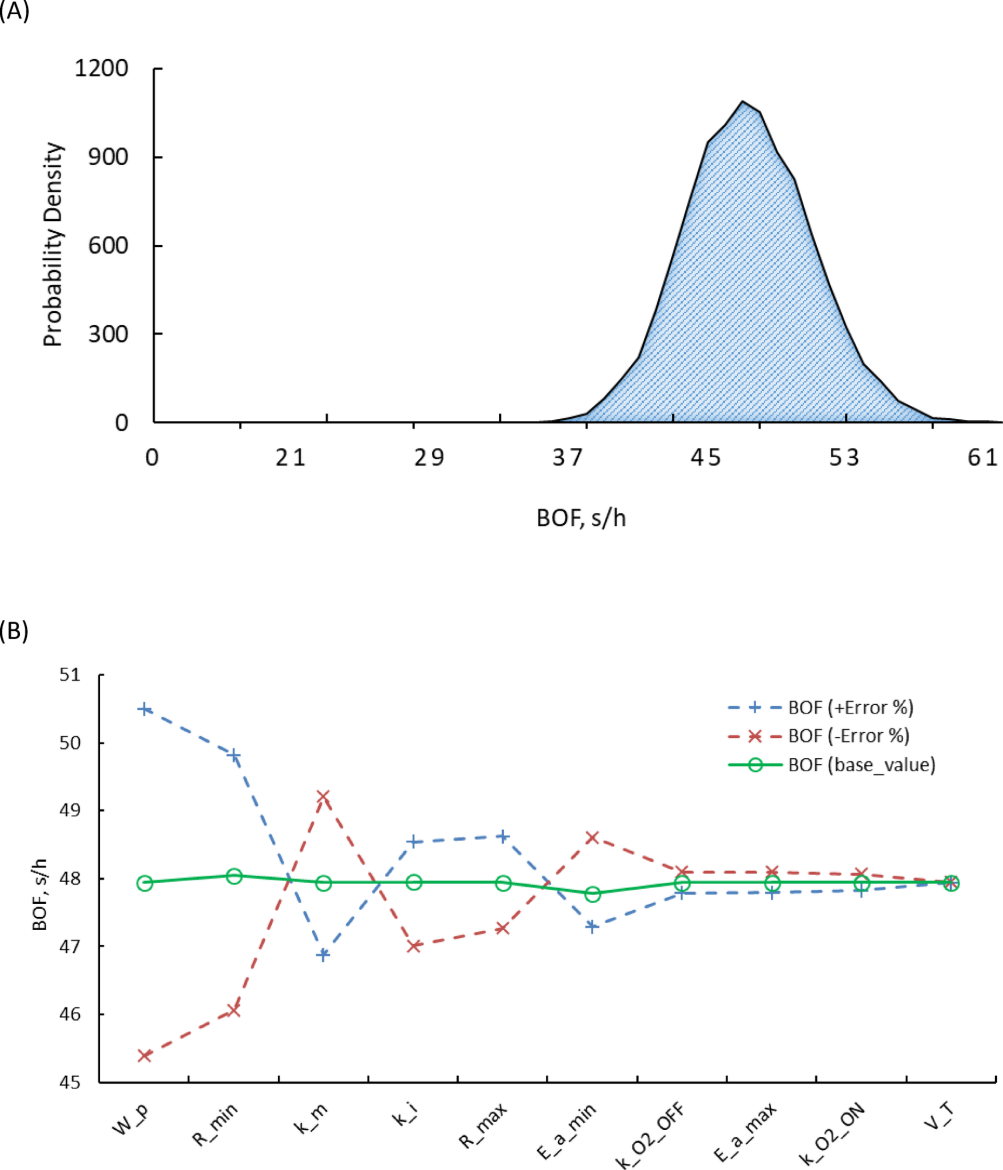
Results of the Monte Carlo simulations (**A**) A probability density plot showing how often different BOF values appeared in the simulations. It highlights the range and variability of BOF values. (**B**) A line graph showing how BOF values change across all simulations. It illustrates the influence of each parameter on the BOF results.

The results shown in Fig. 3 provided key insights into BOF changes under varying parameter conditions. BOF followed a normal distribution with a mean of 47.84s and a standard deviation of 3.69s. This distribution (Fig. 3A) illustrates the variability in BOF due to the combined effect of uncertainties in the model parameters. Sensitivity analysis (Fig. 3B) showed the most important parameters affecting BOF. The weight of the product ($\:{W}_{p}$) was found to be the most effective, with a sensitivity range of 5.11s, the pre-exponential term related to respiration ($\:{R}_{min}$) and the Michaelis constant ($\:{\:k}_{m}$) with ranges of 3.76s and 2.33s, respectively. In contrast, the rest of the parameters showed weaker effects, with sensitivity ranges below 1.5 s, indicating that they had relatively small contributions under the modelled conditions.

**Fig. 4:**
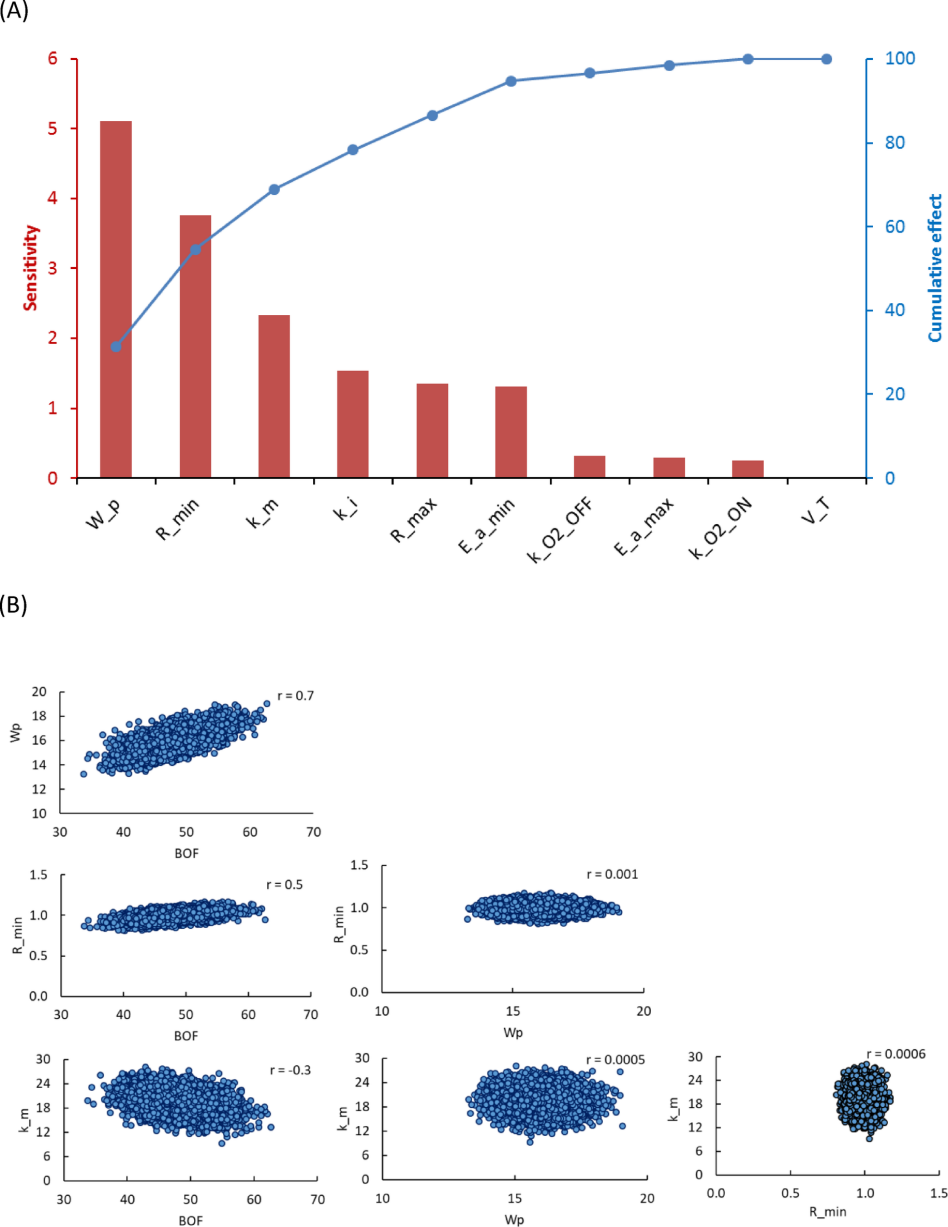
Results of sensitivity analysis with correlation (r) coefficient (**A**) Pareto chart showing the influence of the model parameters on BOF. The cumulative line shows the proportion of the total effect of each parameter, (**B**) Pairwise scatter plot highlighting the relationship and interaction between the top three parameters and BOF.

The Pareto plot of the sensitivity ranking (Fig. 4A) confirmed that$\:\:{W}_{p}$, $\:{R}_{min}$ and$\:{\:k}_{m}$ together accounted for over 80% of the BOF variability, highlighting them as important factors to prioritize for the gas control optimization. The graph also showed the cumulative sensitivity percentage, with the 80% threshold reached by these three parameters, as a result, the most influential parameters were identified for prioritization. This prioritization was in line with the principles of the 80/20 rule, allowing targeted refinement efforts in the modelling and experimental setups. The 80% contribution threshold is consistent with the Pareto principle, which suggests that a small subset of variables accounts for the majority of variability in a model. By focusing on the top 20% of influential parameters, a more efficient and applicable mathematical model can be developed. This approach captured most of the variability in the system and minimized the complexity of dealing with less influential parameters. The remaining 20% of parameters contribute little to the overall variability and are less important for the model.

The results of the sensitivity analysis showed in the pairwise scatter plots (Figure S1), highlighted the relationships and interactions between all model parameters and BOF. The interactions between BOF, and the parameters, such as $\:{W}_{p},\:{R}_{min}\:and\:{\:k}_{m}\:$showed a meaningful correlation with each other, than the other parameters. From the scatter plots (Fig. 4B), the correlation analysis showed a moderate to strong positive relationship between BOF and $\:{W}_{p}$ (*r* = 0.7), indicating that as the product weight increases, the BOF tends to increase. Similarly, a moderate positive correlation was observed between BOF and $\:{R}_{min}$ (*r* = 0.5), indicating that higher values of $\:{R}_{min}$ are associated with increased blower operation. In contrast, BOF and $\:{\:k}_{m}\:$showed a weak negative correlation (*r* = -0.3), indicating that higher values of $\:{\:k}_{m}\:$are associated with a slight decrease in BOF. The rest of the parameters like blower diffusion coefficients ($\:{k}_{{\text{O}}_{{2}_{\text{O}\text{N}}}},\:{k}_{{\text{O}}_{{2}_{\text{O}\text{F}\text{F}}}})\:$and activation energy ($\:{{E}_{a}}_{max}$,$\:{{E}_{a}}_{min}$) exhibit weaker or no meaningful linear relationship with BOF. The interaction analysis of the top three parameters showed that the complex dynamics between the parameters were present. For example, the combined effects of $\:{R}_{min}\:$ and $\:{\:k}_{m}$ increased the variability of the BOF, indicating that they have an additive effect on BOF variability. Similarly, interactions between BOF and $\:{R}_{min}\:$ became prominent under high product weight ($\:{W}_{p}$) conditions. These interactions highlighted the importance of considering non-linear and interaction terms in future models that aim to predict BOF under varying operational conditions. The results are consistent with established methodologies, such as those outlined by Saltelli et al.^27^, which highlight the importance of integrating sensitivity analysis into complex systems modelling. Furthermore, the use of the Monte Carlo simulations in agricultural and post-harvest research^28,29^, provides a robust framework for improving modified atmosphere storage systems.

### Variability analysis in O_2_ concentration

**Fig. 5:**
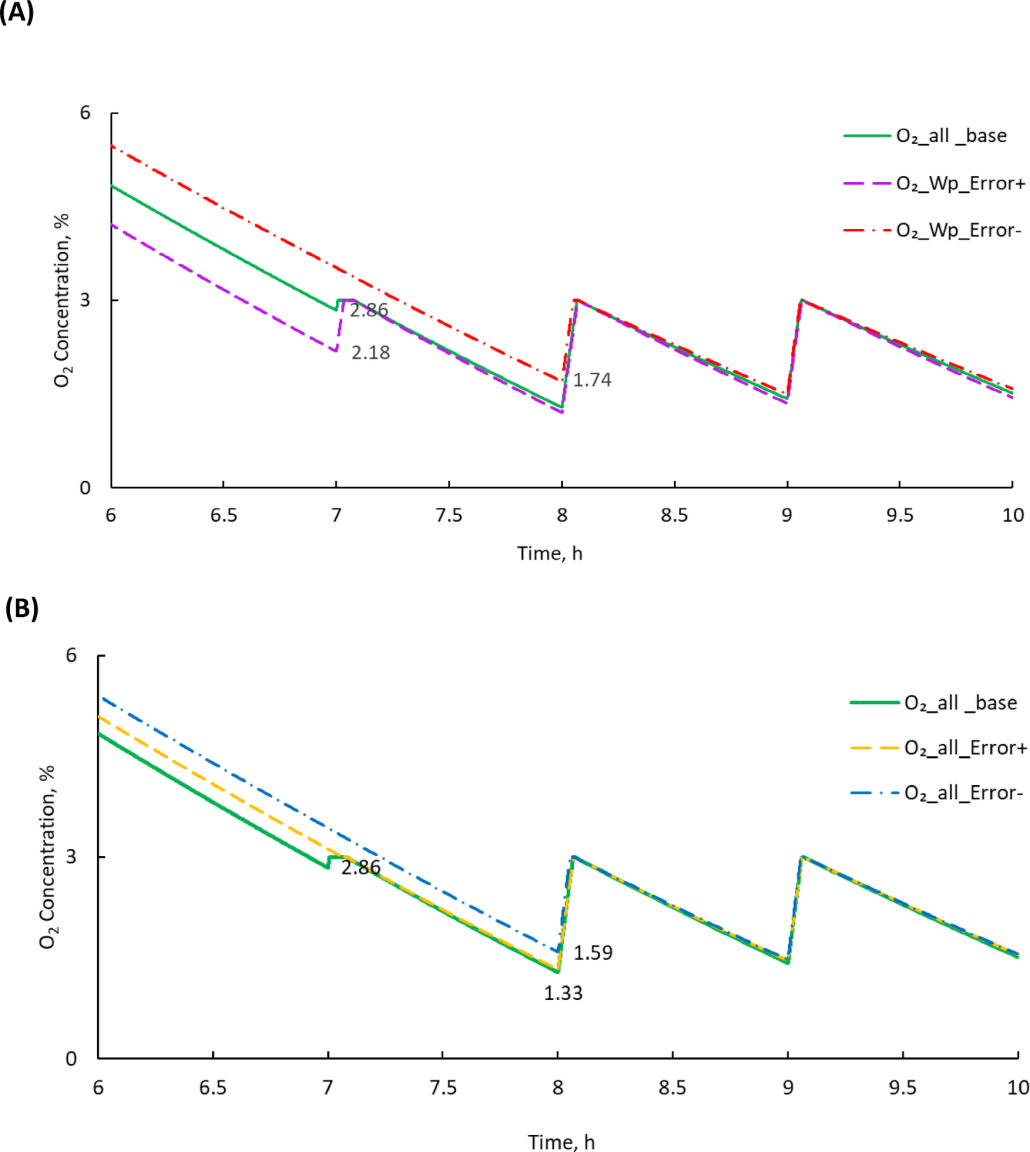
Effect of parameter variability on the O_2_ change (**A**) One variable changed at a time, example zoomed portion of mass variability (**B**) All variable changed at a time, zoomed portion of variability when all variables were considered. The lines show the change in O_2_ concentration for different errors of the model parameters, with the peaks and troughs representing the blower ON and OFF cycles after the 3% O_2_ set point was reached.

Figure 5 illustrates the effect of a single parameter (e.g. mass varied from 15.2 to 16.8 kg from the base value of 16 kg) on the predicted O_2_ concentration along with the BOF and temperature profile during the experiment, with a closer look at the first 10 h for clarity (Fig. 5A). The results showed that when a positive variation in the weight was introduced, the O_2_ concentration reached 3% within 7 h, whereas with a negative variation, it took 8 h for the O_2_ concentration to reach 3%. This was because the O_2_ consumption inside the airtight box was increased due to the higher mass of broccoli. Furthermore, the O_2_ concentration level at the time when the blower turned ON initially, dropped below 3% and reached 1.6% in the case of negative variation and 2.1% in the case of positive variation. But once the blower was initiated, in both cases the O_2_ concentration was effectively maintained at an average of 2.9 ± 0.2% for negative variation and 2.8 ± 0.3% for positive variation. It was found that the drop in O_2_ concentration level due to parameter variation was only at the initial time, until when the blower turned ON, and thereafter it maintained close to the set point.

Figure 5B shows that when variabilities were introduced considering all parameters at a time, the set point for O_2_ concentration was reached after 8 h. The O_2_ concentration at the time when the blower turned ON was dropped to 1.5% for negative variations and then it was maintained at 2.9 ± 0.2%. Similarly, in the case of positive variations, it took 7 h to drop O_2_ concentration to 2.4% and then maintained at 2.7 ± 0.3% as compared to the mean O_2_ concentration estimated with base values at 2.9 ± 0.2%. The data showed that the drop in O_2_ concentration level due to variation in all parameters (Fig. 5B) followed the same trend as of variation due to a single parameter shown in Fig. 5A.

**Fig. 6:**
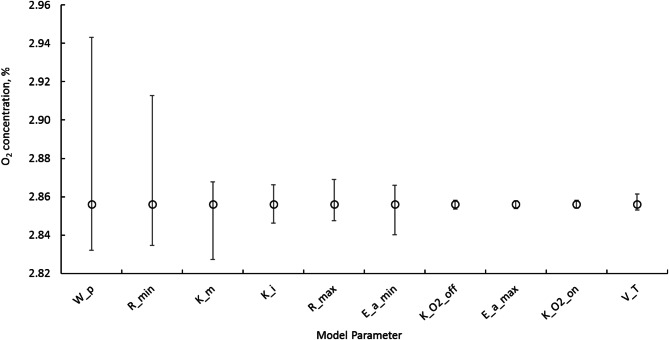
The effect of sensitivity in model parameters on O_2_ concentration analysed with a one-parameter-at-a-time approach. The plot shows the sensitivity of the O_2_ concentration (%) to different model parameters, the error bars reflect how fluctuations in each parameter affected the O_2_ concentration, showing the spread or range of change in O_2_ concentration. Smaller error bars indicate the minimal impact on the O_2_ concentration.

Analysing the absolute differences between means of O_2_ concentrations calculated using base values and those derived from both positive and negative variations, provided insights into understanding each parameter’s importance in maintaining the O_2_ concentration (Fig. 6). The observed differences in means of O_2_ concentration highlighted the varying sensitivities of key model parameters. The parameters like$\:\:{W}_{p}$, $\:{R}_{min}$ and $\:{\:k}_{m}$ showed relatively larger differences, indicating their significant impact on O_2_ concentration regulation and thus higher sensitivity to O_2_ variations. For example, the variations in $\:{W}_{p}$ changed O_2_ concentration from 2.9 to 2.8% and in $\:{R}_{min}$ showed the O_2_ concentration from 2.9 to 2.8% when negative and positive variations were introduced respectively. These variations emphasize the importance of these parameters in maintaining O_2_ concentration in the storage box. Other parameters like $\:{{E}_{a}}_{max}$, $\:{k}_{{\text{O}}_{{2}_{\text{O}\text{N}}}}$, and $\:{k}_{{\text{O}}_{{2}_{\text{O}\text{F}\text{F}}}}$ exhibited relatively insignificant differences which indicate that variation among their values does not make a stronger impact on the effective management of O_2_ but the presence of these parameters were required for the application of the developed model to MA box for fresh produce.

### Experimental validation of BOF and O_2_ concentration

**Fig. 7:**
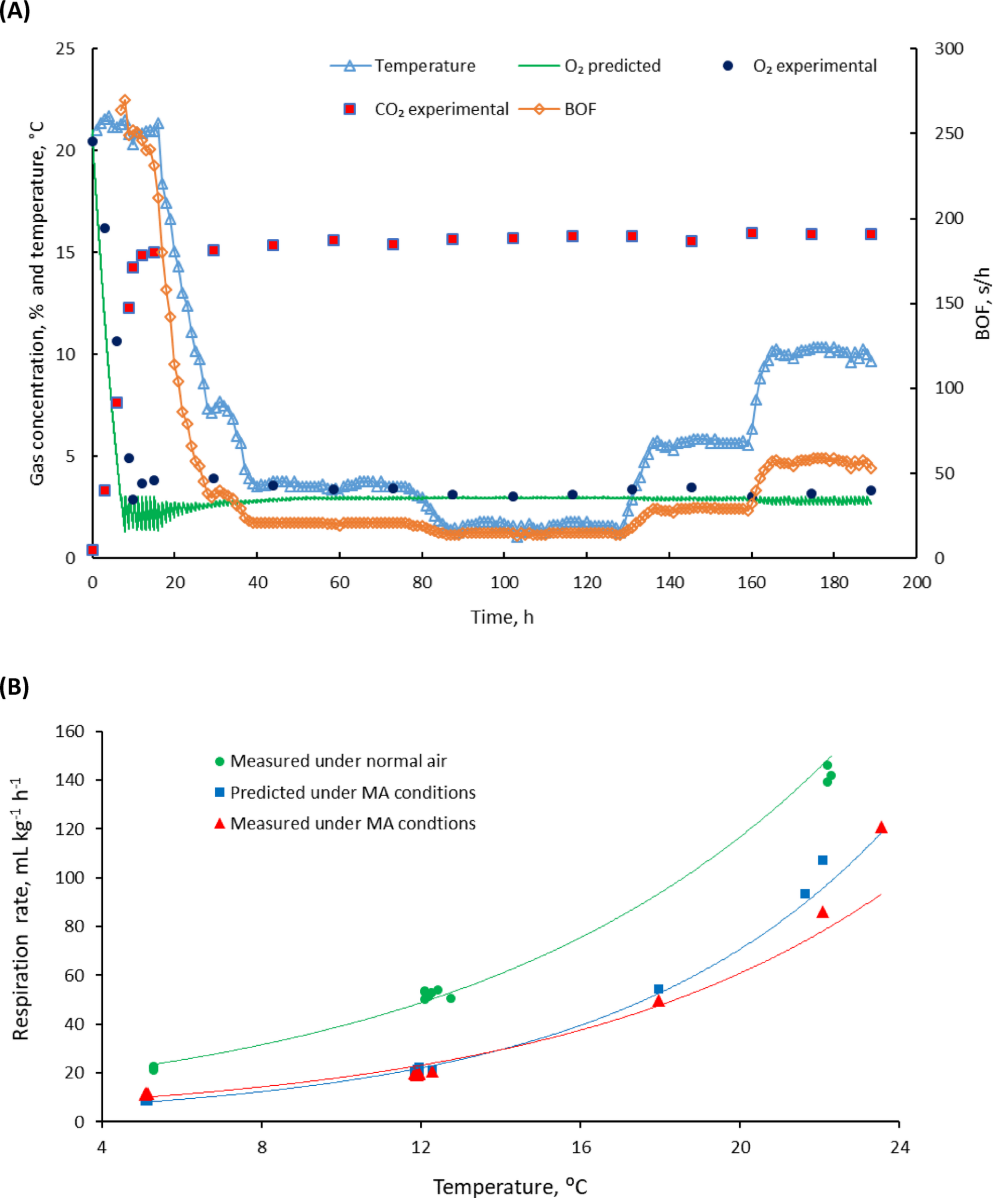
Experimental results to verify model performance under variable temperature conditions for broccoli (**A**) The figure shows the time evolution of O_2_ concentration (experimental and predicted), experimental CO_2_ concentration, temperature and BOF demonstrating the performance of the model under variable temperature conditions, with O_2_ and CO_2_ concentrations stabilized after initial O_2_ pulldown. The BOF adjustments correspond to the temperature variations to maintain the O_2_ concentration. (**B**) Experimental and predicted respiration rate of broccoli for the temperature profile over the experimental duration.

The results (Fig. 7A) showed that the developed model for estimation of BOF effectively maintained the O_2_ concentration at the set point of 3% for a 70 L storage box containing 16 kg broccoli. The initial rapid decline in O_2_ concentration was attributed to the high respiration of broccoli^14,30^ and the absence of blower operation during this phase. Figure 7B represents the predicted and experimental respiration rate of broccoli with different temperatures and atmospheric conditions. From the figure, it is clear that the respiration rate of broccoli was higher in fresh air conditions compared to MA conditions, and it varied with temperature in both fresh air and MA environments^31^. The respiration rate of broccoli at 20 °C was found to be 141.9 mL kg^− 1^ h^− 1^ in air and 120.9 mL kg^− 1^ h^− 1^ in the modified atmosphere (3% O_2_ and 16% CO_2_). The measured values of respiration rates (R² = 0.96) from the experiment showed close matching with predicted values (R² = 0.99) under MA conditions. The average difference between measured and predicted respiration rate was found to be 2.1% which was below the 5% error or 95% level of confidence.

It was also found that fluctuations in O_2_ concentration (Fig. 7A) due to blower activation cycles per hour were more pronounced at higher temperatures than at lower temperatures e.g. 1.5 to 3.0% at 20 °C and 2.8 to 3.0% at 5 °C. The blower was first activated 7 h after the experiment commenced, at which point the O_2_ concentration had decreased to 2.7%. Following blower activation, the O_2_ concentration was stabilized to 2.9 ± 0.2% through subsequent ON-OFF cycles. The correlation analysis of parameters within the 0 to 12.5 °C temperature range highlights key relationships among temperature, BOF, respiration rates and gas composition. Temperature strongly correlated with BOF (R^2^ = 0.99) and respiration rates (R^2^ = 0.97), indicating increased temperatures significantly elevated BOF and respiration rates. This aligns with the known biological response of enhanced metabolic activity at higher temperatures. BOF and respiration rates also exhibit a very strong positive correlation (R^2^ = 0.99), reinforcing the critical influence of respiration dynamics on blower operations.

On the other hand, O_2_ and CO_2_ concentrations showed weaker correlations with temperature, proving that the model concept of controlling gas composition to the set point worked despite temperature changes. If there is weight change, sensitivity analysis proved that a ± 5% variation does not impact the model’s ability to maintain the O_2_ set point. However, if the variation exceeds 5%, it is recommended to rerun the model to adjust its parameters in real-time. The experimental data for O_2_ and CO_2_ confirmed that the model, coupled with the electronic gas control system, successfully maintained the optimal storage conditions for the broccoli throughout the experimental period. Furthermore, the study demonstrated the efficacy of BOF cycles in stabilizing O_2_ concentrations despite temperature fluctuations. Literature supports the notion that temperature variations can influence respiration rates and gas dynamics within the packaging, necessitating adaptive control systems for optimal results^32,33^. These findings not only corroborate existing evidence on the benefits of MAP in fresh produce storage^34,35^ but also highlight the potential for advanced modelling techniques to refine gas exchange mechanisms^31,36–39^ thereby improving storage outcomes for fruit and vegetables.

The developed model was successful in controlling the O_2_ concentration inside the broccoli storage box, despite some variabilities in the model parameters and temperature fluctuations. Although CO_2_ was not controlled by the model directly, experimental data for CO_2_ (Fig. 7A) showed that it was increased up to 15.3 ± 1% and then reached equilibrium level. This was because, the CO_2_ concentration difference between the inside of the box (15.3%) and the outside (0.04%) was similar to the O_2_ concentration difference between the inside (3.5%) and the outside (21%). Thus, at higher CO_2_ concentrations, CO_2_ was removed at the expense of fresh air with each ON-OFF cycle of the blower, and a steady CO_2_ concentration was maintained. This is an advantage for high CO_2_-tolerant commodities^26,40^ such as broccoli. For low CO_2_ tolerant commodities, the system was improved by integrating a soda-lime-based CO_2_ adsorption reactor with active airflow using an air blower managed by the microcontroller and the CO_2_ absorption kinetics was studied and modelled using the Weibull function^41^. This can be further improved by adjusting the blower ON time dynamically based on temperature variations, product respiration rate and CO_2_ set point so that this work can apply to a wide range of fresh produce to improve the shelf life.

## Conclusion

- The model for estimating the BOF was tested for its parameter variability with experimental validation considering the broccoli supply chain’s temperature profile (1 °C to 20 °C). Sensitivity analysis was performed using Monte Carlo simulations and one-factor-at-time methods. The experimentally determined parameter values were used together with their standard deviations, taking into account the respective absolute or percentage errors for the sensitivity analysis. The analysis provided valuable insight into the variability of BOF values, which followed a normal distribution with a mean of 47.8 ± 3.7 s reflected variability due to uncertainties in model parameters.
- The results of the analysis showed that key parameters, including product weight, pre-exponential factor and Michaelis-Menten constant, were the most influential parameters affecting BOF respectively highlighting the need to prioritize these factors for model robustness. These key parameters together accounted for over 80% of the variability in BOF. This finding is consistent with the 80/20 rule, suggesting that focusing on these parameters could lead to significant improvements in the model performance.
- Interaction analysis showed a strong positive correlation between weight and respiration process, and a strong negative correlation between weight and Michaelis-Menten constant, while other parameters such as diffusion coefficients and activation energies showed weak or no linear relationships.
- The validation experiment showed the effect of parameter variation on BOF and O_2_ concentration, showing that variations in the parameters resulted in small but manageable fluctuations in BOF and thus in O_2_ concentration. These findings demonstrated the robustness of the model in effectively managing variations while ensuring the O_2_ concentration (3.5%) and CO_2_ of 15.3% at the end of the storage period.
- This study demonstrated the feasibility of a model-based dynamic O_2_ control system in modified atmosphere storage even if there is variability in the product, diffusion and storage parameters.

## Electronic supplementary material

Below is the link to the electronic supplementary material.

Supplementary Material 1

## Data Availability

The data will be available from the corresponding author, PMahajan@atb-potsdam.de, upon reasonable request.

## References

[1] Beaudry, R. M. Future trends and innovations in controlled atmosphere storage and modified atmosphere packaging technologies. *Acta Hortic.* **876**, 21–28. 10.17660/ActaHortic.2010.876.1 (2010).

[2] Caleb, O. J., Mahajan, P. V., Al-Said, F. A. J. & Opara, U. L. Modified atmosphere packaging technology of fresh and fresh-cut produce and the microbial consequences—a review. *Food Bioprocess. Technol.* **6**, 303–329. 10.1007/s11947-012-0932-4 (2013). 10.1007/s11947-012-0932-4 PMC7089433 32215166

[3] Büchele, F., Khera, K., Thewes, F. R., Kittemann, D. & Neuwald, D. A. Dynamic control of atmosphere and temperature based on fruit CO_2_ production: Practical application in Apple storage and effects on metabolism, quality, and volatile profiles. *Food Bioprocess. Technol.* **16**, 2497–2510. 10.1007/s11947-023-03079-0 (2023).

[4] Casajús, V., Demkura, P., Civello, M., Lobato, M. G. & Martínez, G. Harvesting at different time-points of day affects glucosinolate metabolism during postharvest storage of broccoli. *Food Res. Int.* **136**, 109529. 10.1016/j.foodres.2020.109529 (2020). 32846593 10.1016/j.foodres.2020.109529

[5] Sousa, A. R., Oliveira, J. C. & Sousa-Gallagher, M. J. Determination of the respiration rate parameters of Cherry tomatoes and their joint confidence regions using closed systems. *J. Food Eng.* **206**, 13–22. 10.1016/j.jfoodeng.2017.02.026 (2017).

[6] Li, L., Lv, F. Y., Guo, Y. Y. & Wang, Z. Q. Respiratory pathway metabolism and energy metabolism associated with senescence in postharvest broccoli (Brassica Oleracea L. Var. italica) florets in response to O_2_/CO_2_ controlled atmospheres. *Postharvest Biol. Technol.* **111**, 330–336 (2016).

[7] Oliveira, J. C., Ramos, A. V. & Sousa-Gallagher, M. J. A meta-study of the permeance of perforated packaging films to oxygen and carbon dioxide. *Food Eng. Rev.* **14**, 328–352. 10.1007/s12393-019-09202-2 (2022).

[8] Nur Hanani, Z. A., Sonawane, A. D. & Mahajan, P. V. Impact of humidity, temperature and condensation on O_2_ and CO_2_ transmission rate of modified atmosphere packages. *Food Packag. Shelf Life*. **39**, 101132. 10.1016/j.fpsl.2023.101132 (2023).

[9] Malcolm, N. F. Jr. & Gilbert, B. H. Automatic fresh air exchange system. *US Patent* 7,089,751 B2 (2006). https://patentimages.storage.googleapis.com/c4/ff/bb/606420564d4a48/US7089751.pdf

[10] Lawson, I. J. Controlled atmosphere and modified atmosphere guidelines: Refrigerated cargo ships and refrigerated containers. *Int. Cold Chain Technol.* 1–14 (2014). https://www.crtech.co.uk/pages/ICCT/ICCT_CA_MA_Guidelines_May_2014.pdf

[11] Jo, Y. H., Kim, N. Y., An, D. S., Lee, H. J. & Lee, D. S. Modified atmosphere container equipped with gas diffusion tube automatically controlled in response to real-time gas concentration. *Biosyst. Eng.* **115**, 250–259. 10.1016/j.biosystemseng.2013.03.014 (2013).

[12] Jo, Y. H., An, D. S. & Lee, D. S. Active air Flushing in a sensor-controlled fresh produce container system to maintain the desired modified atmosphere. *Biosyst. Eng.* **125**, 122–127. 10.1016/j.biosystemseng.2014.07.003 (2014).

[13] Lee, J. H., An, D. S., Song, J. M., Jung, Y. B. & Lee, D. S. An adaptively controlled modified atmosphere container system for fresh produce. *Biosyst. Eng.* **148**, 11–17. 10.1016/j.biosystemseng.2016.05.003 (2016).

[14] Lee, J. H., An, D. S. & Lee, D. S. Fresh produce container adaptively controlled in its atmosphere modification under variable temperature conditions. *Biosyst. Eng.* **171**, 265–271. 10.1016/j.biosystemseng.2018.05.005 (2018).

[15] Savur, S., Magali, C., Lee, J. & Rodney, J. Apparatus and methods for controlling atmospheric gas composition within a container. *WO 2012/149611 A1* (2012). https://patentimages.storage.googleapis.com/b1/14/d4/06183d314b76d9/WO2012149611A1.pdf

[16] Savur, S., Linde, G., Rodney, J., Lee, J. & Barker, L. Method for controlled venting of chamber. *US 009032867 B2* (2015). https://patentimages.storage.googleapis.com/ab/c3/73/9b702da8735982/US9032867.pdf

[17] Savur, S., Lee, J. & Rodney, J. Monitoring state of produce within transport containers. *EP 3328749 B1* (2020). https://patentimages.storage.googleapis.com/92/cf/ef/a2e5b6df74cd73/EP3328749B1.pdf

[18] Alzuabi, R. O., Hassan, N. M. & Bahroun, Z. Development and implementation of an innovative smart storage system for fruit quality preservation. *Food Bioprocess. Technol.* **17**, 257–270. 10.1007/s11947-023-03132-y (2024).

[19] Keshri, N. et al. Development of a controlled-ventilation box for modified-atmosphere storage of fresh produce. *Foods* **10**, 2965. 10.3390/foods10122965 (2021). 34945516 10.3390/foods10122965 PMC8701251

[20] Jalali, A., Linke, M., Weltzien, C. & Mahajan, P. Developing an Arduino-based control system for temperature-dependent gas modification in a fruit storage container. *Comput. Electron. Agric.* **198**, 107126. 10.1016/j.compag.2022.107126 (2022).

[21] Kalnar, Y. B., Jalali, A., Weltzien, C. & Mahajan, P. Mathematical model and electronic system for real-time O_2_ control in storage boxes: development and validation under fluctuating temperatures. *Biosyst. Eng.* **242**, 67–79. 10.1016/j.biosystemseng.2024.04.012 (2024).

[22] Seefeld, H. S., Løkke, M. M. & Edelenbos, M. Effect of variety and harvest time on respiration rate of broccoli florets and wild rocket salad using a novel O_2_ sensor. *Postharvest Biol. Technol.* **69**, 7–14. 10.1016/j.postharvbio.2012.01.010 (2012).

[23] Arrhenius, S. Über die dissociationswärme und Den einfluss der temperatur auf Den dissociationsgrad der elektrolyte. *Z. Phys. Chem.* **4** (1), 96–116. 10.1515/zpch-1889-0408 (1889).

[24] Fernandez-Leon, M. F., Fernandez-Leon, A. M., Lozano, M., Ayuso, M. C. & Gonzalez-Gomez, D. Altered commercial controlled atmosphere storage conditions for ‘parhenon’ broccoli plants (Brassica Oleracea L. Var. italica). Influence on the outer quality parameters and on the health-promoting compounds. *LWT-Food Sci. Technol.* **50**, 665–672. 10.1016/j.lwt.2012.07.028 (2013).

[25] Jurij, N., Beno, S., Matjaz, G. & Drago, B. Sensitivity analysis of computational models for solving paradox. *J. Netw. Technol.* **10** (1), 18–24. 10.6025/jnt/2019/10/1/18-24 (2019).

[26] Cantwell, M., Suslow, T. & Broccoli Recommendations for maintaining postharvest quality. *University of California, Davis*. https://postharvest.ucdavis.edu/produce-facts-sheets/broccoli (Accessed on March 25, 2024).

[27] Saltelli, A., Tarantola, S. & Campolongo, F. Sensitivity analysis as an ingredient of modeling. *Stat. Sci.* **15** (4), 377–395 (2000). http://www.jstor.org/stable/2676831

[28] Templalexis, C. G. & Xanthopoulos, G. T. Monte Carlo simulation and sensitivity analysis of the Michaëlis-Menten kinetic equation for the CO_2_ Inhibition response to O_2_ consumption during storage of fresh produce. *Biosyst. Eng.* **232**, 129–140. 10.1016/j.biosystemseng.2023.07.005 (2023).

[29] Ding, C., Shuning, S., Jianjun, C., Wei, W. & Zuojun, T. Analysis of light transport features in stone fruits using Monte Carlo simulation. *PLoS One*. **10**, e0140582. 10.1371/journal.pone.0140582 (2015). 26469695 10.1371/journal.pone.0140582 PMC4607418

[30] Belay, Z. A., Caleb, O. J. & Opara, U. L. Influence of initial gas modification on physicochemical quality attributes and molecular changes in fresh and fresh-cut fruit during modified atmosphere packaging. *Food Packag. Shelf Life*. **21**, 100359. 10.1016/j.fpsl.2019.100359 (2019).

[31] Lee, D. S., Jo, Y. H., Kwon, M. J. & An, D. S. Strategy and software application of fresh produce package design to attain optimal modified atmosphere. *Math. Probl. Eng.* 363691 (2014). (2014). 10.1155/2014/363691

[32] Brecht, J. K., Pliakoni, E. D. & Batziakas, K. The impact of temperature on atmosphere requirements and effects: the limits of design and utility for CA/MA/MAP. In *Controlled and Modified Atmospheres for Fresh and Fresh-Cut Produce* (eds. Gil, M. I. & Beaudry, R.) 147–166. (Academic Press, 2020). 10.1016/B978-0-12-804599-2.00009-0

[33] Beaudry, R. M., Cameron, A. C., Shirazi, A. & Dostal-Lange, D. L. Modified atmosphere packaging of blueberry fruit: Effect of temperature on package O_2_ and CO_2_. *J. Am. Soc. Hortic. Sci.* **117**, 436–441. 10.21273/JASHS.117.3.436 (1992).

[34] Jalali, A., Linke, M., Geyer, M. & Mahajan, P. Integrative programming for simulation of packaging headspace and shelf life of fresh produce. *MethodsX* **8**, 101514. 10.1016/j.mex.2021.101514 (2021). 34754785 10.1016/j.mex.2021.101514 PMC8563652

[35] Chen, X., Xu, C. & Mir, N. Success stories for CA/MA. In *Controlled and Modified Atmospheres for Fresh and Fresh-Cut Produce* (eds. Gil, M. I. & Beaudry, R.) 277–289 (Academic Press, 2020). 10.1016/B978-0-12-804599-2.00014-4

[36] Hertog, M. L. A. T. M., Uysal, I., McCarthy, U., Verlinden, B. M. & Nicolaï, B. M. Shelf life modelling for first-expired-first-out warehouse management. *Philos. Trans. R Soc. Math. Phys. Eng. Sci.* **372**, 20130306. 10.1098/rsta.2013.0306 (2014). 10.1098/rsta.2013.0306 PMC4006170 24797134

[37] Cagnon, T., Méry, A., Chalier, P., Guillaume, C. & Gontard, N. Fresh food packaging design: A requirement driven approach applied to strawberries and agro-based materials. *Innov. Food Sci. Emerg. Technol.* **20**, 288–298. 10.1016/j.ifset.2013.05.009 (2013).

[38] Mahajan, P. V., Oliveira, F. A. R., Montanez, J. C. & Frias, J. Development of user-friendly software for design of modified atmosphere packaging for fresh and fresh-cut produce. *Innov. Food Sci. Emerg. Technol.* **8**, 84–92. 10.1016/j.ifset.2006.07.005 (2007).

[39] Hertog, M. L. A. T. M. & Banks, N. H. Improving modified atmosphere packaging through conceptual models. In* Food Process Modelling* (eds Tijskens, L. M. M. & Hertog, M. L. A. T. M. & Nicolaï, B. M.) 288–311 (Woodhead Publishing, Cambridge, UK, 2001).

[40] Saltveit, M. E. Is it possible to find an optimal controlled atmosphere? *Postharvest Biol. Technol.* **27** (1), 3–13. 10.1016/S0925-5214(02)00184-9 (2003).

[41] Kalnar, Y. B., Sonawane, A. D., Weltzien, C. & Mahajan, P. V. Evaluating soda-lime adsorption and microcontroller-based system for the dynamic control of CO_2_ in a fresh produce storage box under varying temperature. *J. Agric. Food Res.* **21**, 101852. 10.1016/j.jafr.2025.101852 (2025).

