# Supplementary material for: Evaluating the impact of parameter variability on O2 control and model robustness in modified atmosphere storage for fresh produce
Source: Sci Rep. 2025 May 12;15:16487. doi: 10.1038/s41598-025-99854-4 (PMC12069672; doi:10.1038/s41598-025-99854-4)
Supplement: Supplementary file 1 — Supplementary Material 1 [file 41598_2025_99854_MOESM1_ESM.docx]

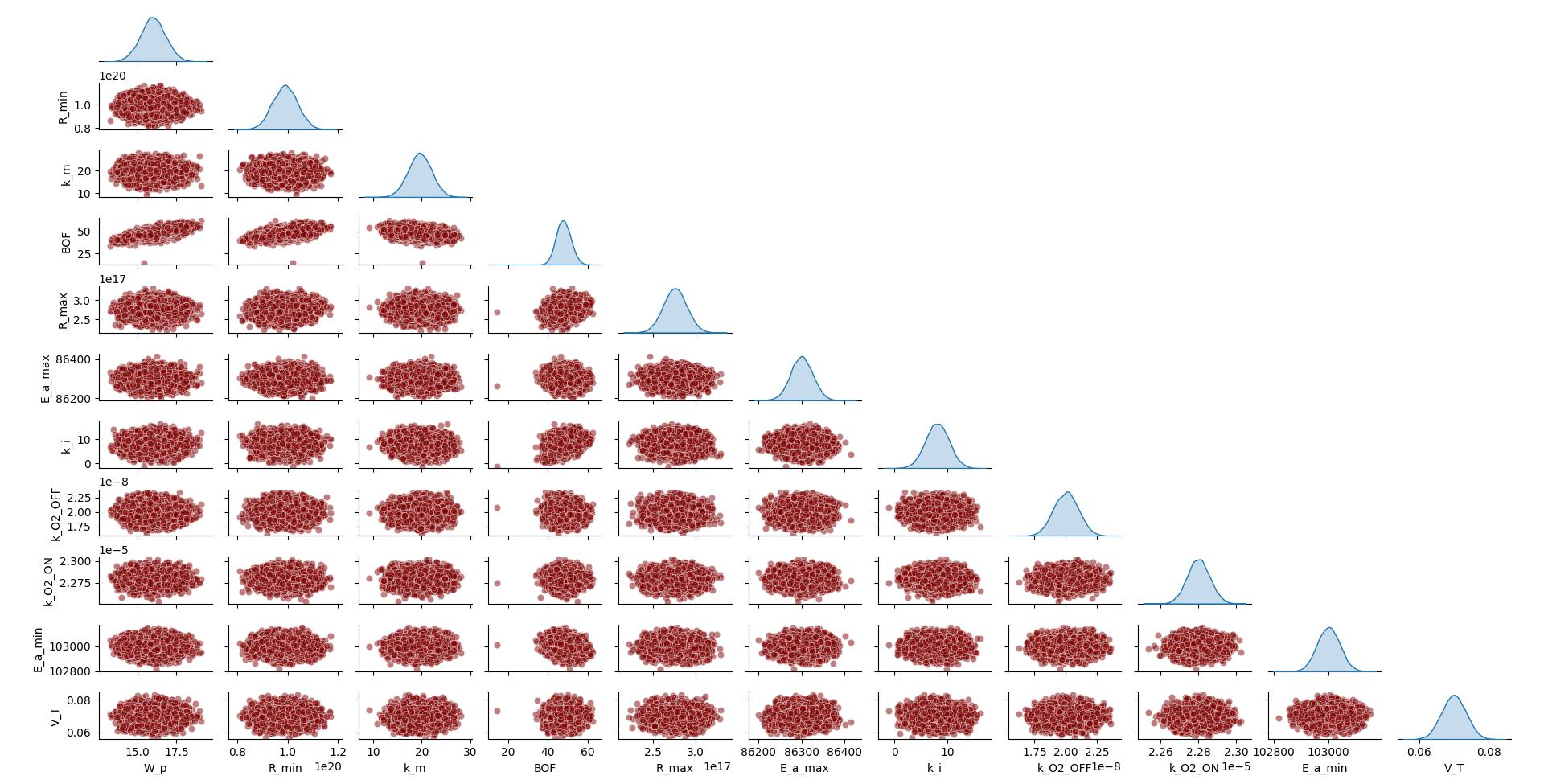


Figure S1. Sensitivity analysis results showing the pairwise scatter plots highlighting the relationship and interaction between all model parameters and Blower ON Time (BOF). The interactions between BOF and the parameters such as $W_{p}, R_{min} and k_{m}$showed a meaningful correlation with each other, than the other parameters.
